# Evaluation of online educational curriculum on HPV vaccination practices among adult primary care providers

**DOI:** 10.1186/s12909-023-04807-y

**Published:** 2023-12-05

**Authors:** Christiana Zhang, Judy Greengold, Sean Tackett, Caroline Lentz, Wendy Bennett, Maura McGuire

**Affiliations:** 1 Division of General Internal Medicine, Johns Hopkins University School of Medicine, Baltimore, USA; 2 Johns Hopkins Community Physicians, Baltimore, USA; 3 Division of General Internal Medicine, Johns Hopkins Bayview Medical Center, Baltimore, USA

**Keywords:** HPV, Vaccination, Medical education, Prevention, Cancer, Online educational module

## Abstract

**Background:**

Human papillomavirus (HPV) is the most common sexually transmitted infection in the United States. While HPV is a vaccine-preventable illness, vaccine utilization rates in the United States remain low, particularly among adults.

**Methods:**

The objective of this study was to assess the impact of an online, asynchronous educational module on HPV vaccination for adult primary care providers. We designed and implemented the module for family medicine, internal medicine, medicine/pediatrics, and obstetrics/gynecology providers in a community practice network affiliated with a large academic health system. We evaluated the effect of the module on provider knowledge, attitudes, and self-reported behaviors with pre-, post-, and delayed post-tests, using Likert-scales for measurement. We summarized data with descriptive statistics and compared changes in individuals using paired t-tests.

**Results:**

One hundred forty-four out of 223 providers completed the module (response rate of 65%). At baseline, internists had the lowest knowledge scores compared to other specialties (pre-test mean of 3.6, out of 5, SD 1.2). Internists were also the least likely to counsel patients on HPV vaccination (mean 1.6, SD 0.9). There was a statistically significant improvement in knowledge from pre-test to post-test (from mean of 3.8 to 4.6, out of 5, *p* < .001) across all specialties. There was also statistically significant improvement in mean confidence for all providers from pre-test to post-test to identify patients aged 19–26 (3.3 to 3.7, *p* < .001) and patients aged 27–45 (2.7 to 3.5, *p *< .001) who needed vaccination. There was a statistically significant improvement in likelihood to counsel eligible patients on the risks of HPV infection (mean 2.3 to 2.8, *p*-value 0.002). The delayed post-test demonstrated retention of improved knowledge, confidence, and self-reported behavior.

**Conclusions:**

This study demonstrated that an asynchronous online module was effective at improving confidence, knowledge, and self-reported behavior of adult primary care providers in recommending HPV immunization. Given the important role that healthcare providers play in vaccine uptake, this study suggests that an online educational intervention can be a powerful tool to encourage increased utilization and delivery of the HPV vaccine. Further efforts are needed to educate internists and providers who take care of the adult population on HPV vaccination.

## Background

Human papillomavirus (HPV) is the most common sexually transmitted infection in the world [1]. In the United States, about 79 million people are infected with HPV and 14 million new infections occur every year [2]. Though most HPV infections are asymptomatic and often clear spontaneously, HPV is associated with multiple medical conditions, including genital warts, anogenital cancers (cervical, vaginal, vulvar, penile, and anal), and oropharyngeal cancers. Approximately 47,199 new cases of HPV-associated cancers occurred each year in the United States from 2015 to 2019 and the majority could be prevented by vaccination [3].

Though there is no antiviral treatment for HPV infection, the nine-valent HPV vaccine (9vHPV) is distributed in the United States for prevention of HPV and licensed for use in patients aged 9 to 45 years old. Patients are recommended to receive HPV vaccination at age 11 or 12 years but can be given starting at age 9. Catch-up vaccination is recommended through age 26 years for patients who are not fully vaccinated. For patients aged 27 through 45 years who are not fully vaccinated, shared decision-making is recommended [4].

Research shows that HPV vaccination is extremely effective at reducing the prevalence of HPV with concomitant reduction in complications of this illness (https://www.cdc.gov/vaccines/vpd/hpv/hcp/safety-effectiveness.html) [5]. However, despite wide availability and broad insurance coverage of the HPV vaccine, HPV vaccination utilization in the United States remains low. According to data from the 2020 National Immunization Survey-Teen, 58.6% of adolescents aged 13–17 were up to date on HPV vaccination [6]. HPV vaccination rates are even lower in adults aged 18–26. In 2019, only 47% of adults aged 18–26 had ever received HPV vaccination [7]. Furthermore, the U.S. Food and Drug Administration (FDA) approved an expansion of the HPV vaccination for patients 27–45 years of age in 2018. In 2019, the Advisory Committee on Immunization Practices encouraged shared clinical decision-making for adults aged 27–45 who may benefit from HPV vaccination. However, rates of HPV vaccination in adults aged 27–45 remain low. National Health Interview Survey 2017 data showed that, out of 9,744 individuals, only 9.7% of adults aged 27–45 had completed the series (females 15.8%, males 3.2%) [8].

Primary care providers (PCPs) play an important role in promoting the utilization and delivery of the HPV vaccine. HPV vaccine recommendations by PCPs can increase HPV vaccination initiation and completion by three- and ninefold, respectively [9, 10]. However, their perceived barriers can affect HPV vaccination. Surveyed adolescent providers have reported that time, infrequent office visits by patients, difficulty ensuring vaccine series completion, and personal discomfort talking about sexually transmitted infections as barriers to HPV vaccination [11]. Furthermore, HPV knowledge among providers remains low [12]. A recent systematic review by Leung et al. performed in 2019 revealed that knowledge among healthcare providers surrounding HPV vaccination was generally low and corresponded with a low vaccination recommendation rate. Providers also reported low self-confidence in counseling and addressing concerns [12]. Improved education for PCPs is therefore needed to further promote HPV vaccination utilization [13].

To address this knowledge deficit and improve utilization of HPV vaccination, we designed and implemented an educational module on HPV vaccination for providers who provided adult primary care in a community practice network affiliated with a large academic health system. Given the low rates of HPV vaccination in the adult population, we sought to address a practice gap by targeted providers who specifically provided care for adult patients over the age of 18 for additional education. We designed surveys to assess the effect of the module on provider knowledge, attitudes, and self-reported behaviors for HPV vaccination among providers.

## Methods

### Setting and targeted learners

The setting was a community-based primary and specialty care practice network affiliated with a large academic health system, with 45 sites in two states and Washington DC. We targeted clinicians who provide primary care services for adult patients over 18 years of age including internal medicine (IM), medicine/pediatrics (Med/Peds), obstetrics/gynecology (OB/GYN), and family medicine (FM) specialties. All clinical provider types, including medical doctors (MDs), doctors of osteopathic medicine (DOs), nurse practitioners (NPs), certified nurse midwives (CNMs), and physician assistants (PAs), were assigned this educational opportunity. This module offered Maintenance of Certification (MOC) and Continuing Medical Education (CME) credits (0.50 American Medical Association's Physician's Recognition Award Category 1 Credits™).

### Design of the educational content and methods for the module

A team of subject matter experts, in collaboration with an external medical education vendor, led the module design and content development. The module was funded through an unrestricted educational grant. The development process was further informed by the six-step method of curriculum development [14]. The educational module was created as an asynchronous, self-paced module that was designed to take approximately 15 min to complete.

The curriculum content included information on HPV prevalence, transmission, and diseases associated with HPV infection. Next, content on understanding and communicating with patients about the HPV vaccination schedule was presented. The content also included 4 case vignettes which reviewed the following topics: recommended HPV vaccination schedule, HPV vaccination schedule disruptions, HPV vaccination for men, and HPV vaccination for patients aged 27–45 years of age. Pop-out windows offering knowledge checks were interspersed to encourage more active learning and interaction with the module.

There was a special focus on strategies to address patient questions or concerns to help providers engage in shared decision-making [15]. Commonly asked questions with example responses were interspersed throughout the module, and included information on risk of contracting HPV, HPV vaccination efficacy and safety, HPV vaccination dosing, HPV vaccination for patients who are not sexually active, and HPV vaccination for men. Finally, the module also included content on HPV vaccination-specific team-based care and decision support tools embedded in the electronic medical record.

### Implementation

Once content and methodology/pedagogy were finalized, the module was reviewed by the Johns Hopkins Community Physicians Research and Projects Committee, who also served to pilot the items. The module then was posted on the organization’s learning management system and assigned to targeted providers. The final product was reviewed by the clinical practice’s education & training committee and accredited by the Johns Hopkins Office of Continuing Medical Education.

In March 2021, the module was assigned as required learning for all providers and announced via email to an audience of 223 adult (IM, FM, Med/Peds, OB/GYN) providers. Participation incentives of low value gift cards ($5) and education credits were offered to encourage completion of the modules and delayed post-test. Data collection on demographics and behavioral questions for the first 31 completions was partially limited by an administrative error. Demographic data was retroactively obtained, but behavioral data was not available for those participants. In November 2021, the delayed post-test was sent out via email to all providers who completed the module.

### Evaluation methods

Evaluation of the learners included pre-test, post-test, and delayed post-test assessments. We designed a pre-test before completing the module to assess confidence and knowledge surrounding HPV vaccination, with four questions on confidence on counseling of HPV vaccination, based on a Likert scale from 1 (not confident) to 4 (very confident). Knowledge assessment consisted of five multiple choice questions on characteristics of HPV infection and clinical sequelae, indications for HPV vaccination and schedule and strategies to overcome barriers to HPV vaccination. The post-test assessment included identical questions on confidence and knowledge, with three additional behavior questions asking providers to assess how often they discussed or counseled patients on HPV vaccination. The knowledge questions provided instant feedback for each knowledge question. The delayed post-test had identical demographic, confidence, knowledge, and behavior questions.

To obtain CME credit, participants were directed to complete a course evaluation with ten additional questions that were based on a Likert scale from 1 (strongly disagree) to 5 (strongly agree).

### Data analysis

We used descriptive statistics (mean, median and proportion) to understand demographic characteristics of providers. We coded the five knowledge items as correct or incorrect and a total score was the sum of correct items. Individual respondents’ mean total scores for pre- vs post-test and post-test vs delayed post-tests were compared using paired t-tests. We also used paired t-tests to compare mean confidence items (along a 1–4 point Likert-type scale) for each of the four items, pre vs post, and post vs delayed post-test. For items asking percentage of time respondents counseled patients about HPV, we used paired t-tests to compare their responses in the course evaluation to responses in the delayed post-test. Statistical analyses were performed using Stata (StataCorp. 2013. Stata Statistical Software: Release 13. College Station, Texas: StataCorp LP).

### Ethics approval

The Johns Hopkins University School of Medicine Institutional Review Board reviewed and approved this study (IRB number IRB00263263).

## Results

### Respondent characteristics

The module was distributed to 223 providers, and a total of 144 providers completed the module pre-test and post-test knowledge items (65%). Due to an administrative error during the initial module deployment, the demographic and behavioral data for the first 31 participants were not collected. 113 providers (78%) completed the updated module with additional questions on behaviors and demographics. The demographic data for the first 31 participants were subsequently obtained manually. 46 out of 144 total providers (32%) completed the delayed post-test. Of the 144 providers who completed the module, 74% were medical doctors and doctors of osteopathic medicine, 2% were physician assistants, and 24% were nurse practitioners or midwives. With regards to specialty breakdown, 59 (41%) were IM providers, 49 (34%) were FM providers, 20 (14%) were Med/Peds providers, and 16 (11%) were OB/GYNs.

### Assessment of knowledge

Of the 144 total providers who completed the pre-test and post-test, there was a statistically significant improvement in the 5 knowledge items and total score (Table 1).

**Table 1:** Assessment of knowledge with pre-test and post-test

Knowledge		Pre-test	Post-test	*p* values
	N	Mean (SD)	Mean (SD)	
	144	3.9 (1.1)	4.7 (0.5)	< 0.001
OB/GYN	16	4.4 (0.8)	4.8 (0.4)	0.029
Med/Peds	20	4.3 (0.9)	4.9 (0.4)	0.004
Family Medicine	49	3.8 (1.1)	4.7 (0.5)	< 0.001
Internal Medicine	59	3.6 (1.2)	4.6 (0.5)	< 0.001

• Knowledge sum of correct items (possible 5)

When stratified by specialty, OB/GYN providers scored the highest on the pre-test with a mean of 4.4 out of 5 with a standard deviation (SD) of 0.8, followed by Med-Peds providers (mean of 4.3, SD 0.9), then FM providers (mean of 3.8, SD 1.1). IM providers scored the lowest with a pre-test mean of 3.6 (SD 1.2). All specialties showed improvement in knowledge with the immediate post-test (*p* < 0.05).

### Assessment of confidence

At baseline, OB/GYN providers were the most confident in their knowledge of the vaccination schedule (mean 3.4 out of 4, SD 0.7), ability to identify patients aged 19–26 (mean 3.8, SD 0.4), ability to identify patients aged 27–45 (mean 3.5, SD 0.6), and ability to counsel patients on HPV vaccination (mean 3.5, SD 0.5). IM providers were the least confident in their knowledge of the vaccination schedule (mean 2.6, SD 0.8), ability to identify patients aged 19–26 (mean 3.0, SD 0.9), ability to identify patients aged 27–45 (mean 2.3, SD 0.9), and ability to counsel patients on HPV vaccination (mean 2.7, SD 0.8). (Table 2).

**Table 2:** Assessment of confidence with pre-test and post-test

		Pre-test	Post-test	*p* values
Confidence	N	Mean (SD)	Mean (SD)	
1. Ability to identify patients aged 19–26 who need HPV vaccination?	144	3.3 (0.8)	3.7 (0.5)	< 0.001
OB/GYN	16	3.8 (0.4)	3.9 (0.3)	0.333
Med/Peds	20	3.8 (0.4)	3.9 (0.3)	0.163
Family Medicine	49	3.4 (0.6)	3.6 (0.6)	0.002
Internal Medicine	59	3.0 (0.9)	3.6 (0.5)	< 0.001
2. Ability to identify patients aged 27–45 who need HPV vaccination?	144	2.7 (0.9)	3.5 (0.6)	< 0.001
OB/GYN	16	3.5 (0.6)	3.8 (0.4)	0.020
Med/Peds	20	3.0 (0.9)	3.7 (0.6)	< 0.001
Family Medicine	49	2.9 (0.8)	3.5 (0.6)	< 0.001
Internal Medicine	59	2.3 (0.9)	3.4 (0.6)	< 0.001
3. Knowledge of the recommended schedule and timing for HPV vaccination	144	3.0 (0.8)	3.6 (0.5)	< 0.001
OB/GYN	16	3.4 (0.7)	3.7 (0.5)	0.041
Med/Peds	20	3.4 (0.6)	3.9 (0.3)	0.002
Family Medicine	49	3.1 (0.8)	3.6 (0.6)	< 0.001
Internal Medicine	59	2.6 (0.8)	3.5 (0.6)	< 0.001
4. Ability to counsel your patients on HPV vaccination?	144	3.0 (0.8)	3.6 (0.5)	< 0.001
OB/GYN	16	3.5 (0.5)	3.8 (0.4)	0.041
Med/Peds	20	3.4 (0.9)	3.8 (0.4)	0.015
Family Medicine	49	3.2 (0.6)	3.7 (0.6)	< 0.001
Internal Medicine	59	2.7 (0.8)	3.5 (0.5)	< 0.001

• Confidence Variable (1 = not confident, 4 = extremely confident)

There was a statistically significant improvement in overall mean confidence for all providers from pre- to post-assessment in ability to identify patients aged 19–26 (3.3, SD 0.8 vs 3.7, SD 0.5, *p* < 0.001) and patients aged 27–45 (2.7, SD 0.9 vs 3.5 SD 0.6, *p* < 0.001) who needed vaccination. Similarly, there was a statistically significant improvement in confidence for all providers with regards to knowledge of the vaccination schedule (3.0, SD 0.8 vs 3.6, SD 0.5) and ability to counsel patients on HPV vaccination (3.0, SD 0.8 vs 3.6, SD 0.5) (Table 2).

### Baseline behavior

**Fig. 1:**
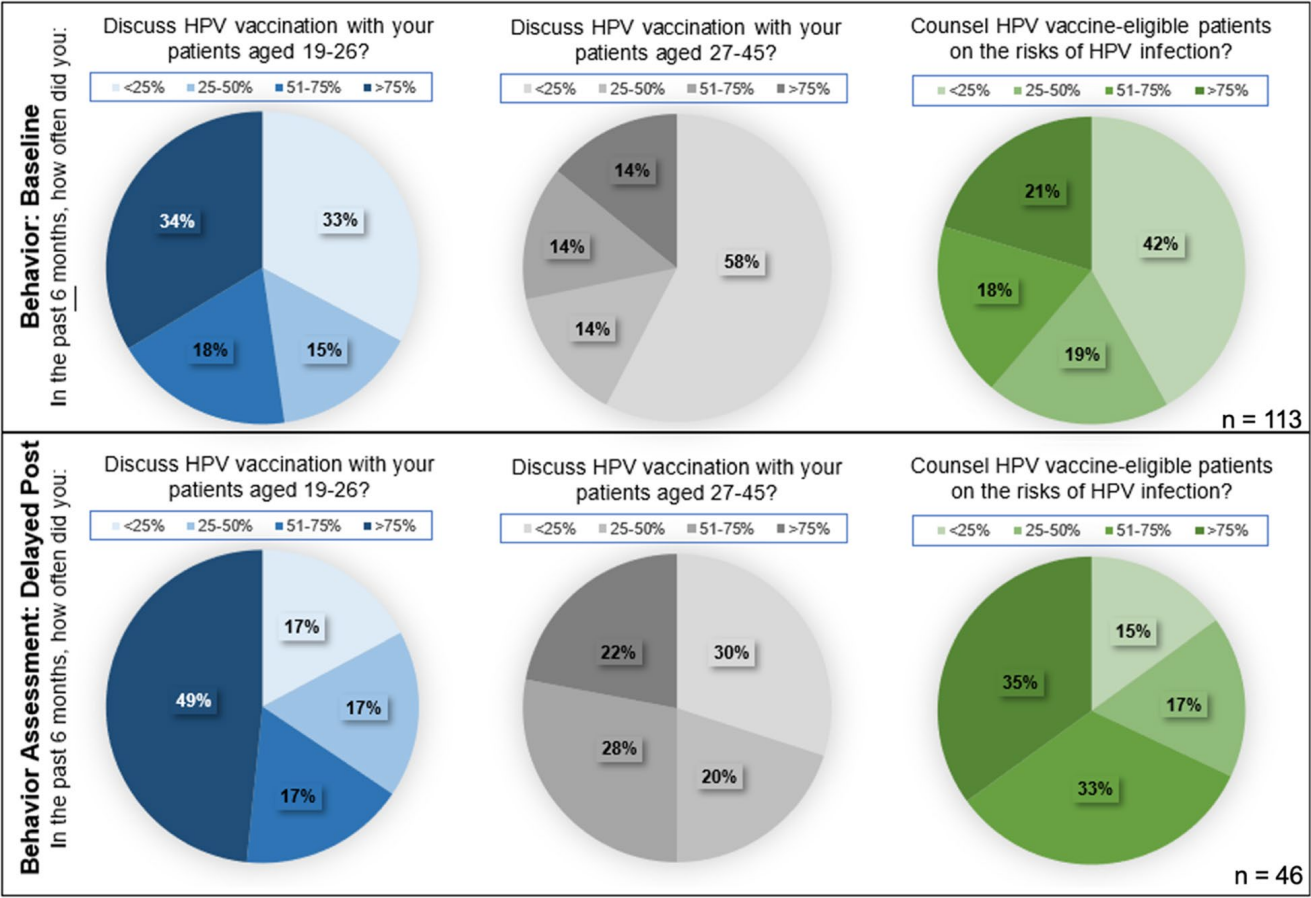
Assessment of self-reported behavior

67% (76/113) of providers discussed HPV vaccination with patients aged 19–26 more than 25% of the time in the past 6 months. In contrast, only 42% (48/113) of providers discussed HPV vaccination with patients aged 27–45 more than 25% of the time. 58% (65/113) of providers reported discussing HPV vaccination with patients aged 27–45 less than 25% of the time. 42% (47/113) of providers reported that they counseled eligible patients on HPV vaccination less than 25% of the time in the last 6 months (Fig. 1).

IM providers were the least likely to discuss HPV vaccination with their patients aged 19–26 (mean 2.2 out of 4, SD 1.2) and patients aged 27–45 (mean 1.4, SD 0.9). They were also the least likely to counsel patients on HPV vaccination (mean 1.6, SD 0.9). OB/GYN providers were the most likely to discuss HPV vaccination with their patients aged 19–26 (mean 3.4 out of 4, SD 0.9) and patients aged 27–45 (mean 2.7, SD 1.1). They were the most likely to counsel patients on HPV vaccination as well (mean 3.2, SD 1.1).

### Assessment of retention of knowledge, confidence, and behavior

**Fig. 2:**
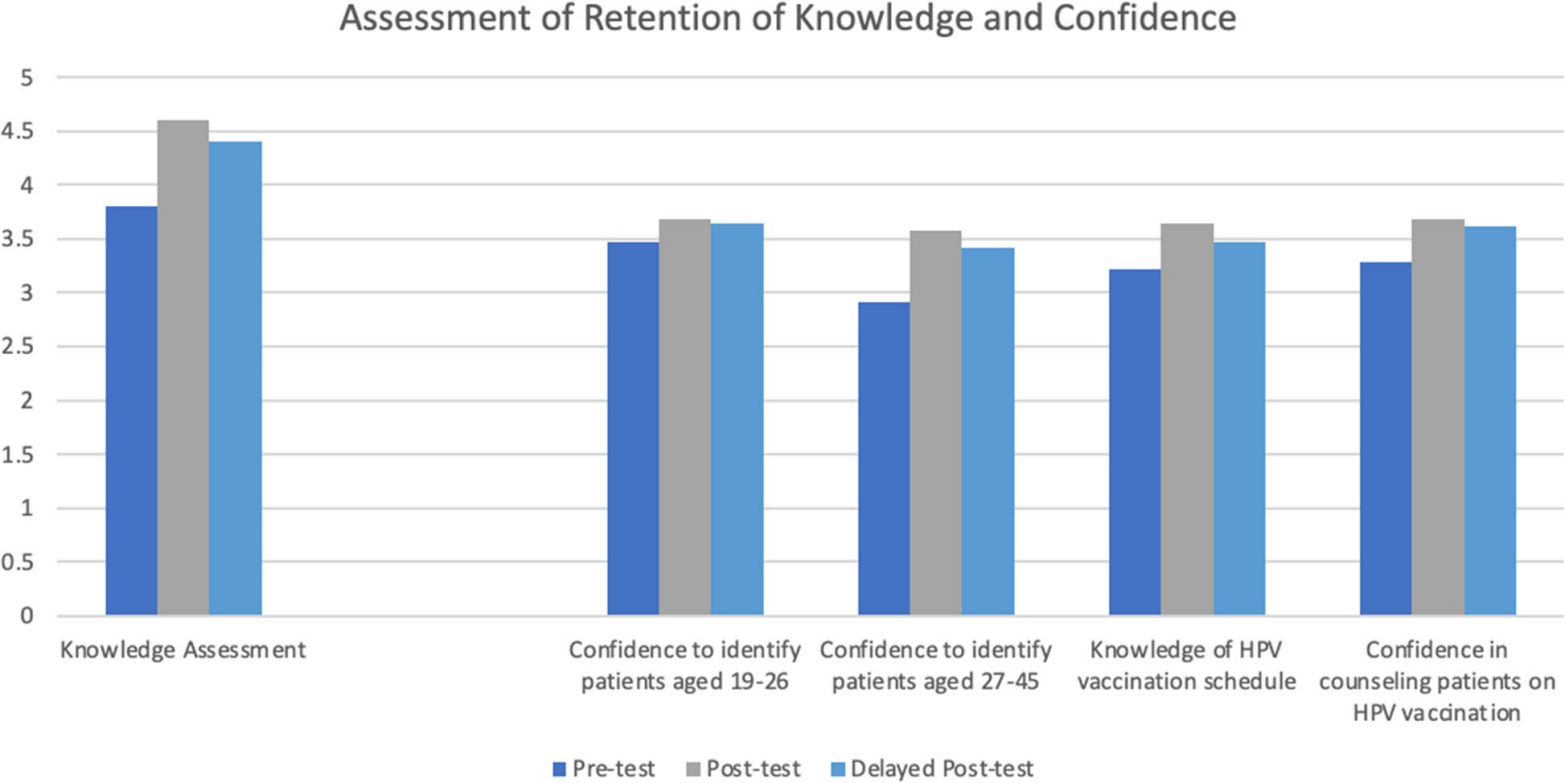
Assessment of retention of knowledge and confidence • Knowledge variable: Sum of 5 knowledge assessment questions. • Confidence variable: 1 = not confident, 4 = extremely confident

Of the 45 providers who completed the delayed post-test, mean scores for knowledge and confidence declined slightly from the post-test to the delayed post-test. The delayed post-test showed sustained improvement in knowledge and confidence compared to the initial pre-test (Fig. 2).

Post-test and delayed post-test behavior data were available for comparison for 34 providers who completed the baseline post-test and the delayed post-test (34/46, 74%). With regards to discussing HPV vaccination with patients aged 19–26, the mean increased from 2.7 from the immediate post-test to 3.0 with the delayed post-test (*p* value = 0.078). Providers discussed HPV vaccination less frequently with patients aged 27–45 compared to patients aged 19–26, but the frequency also improved from the immediate post-test to the delayed post-test (mean increased from 2.1 to 2.4, *p*-value = 0.039). There was a statistically significant improvement in the frequency of counseling eligible patients on the risks of HPV infection (mean improved from 2.3 to 2.8, *p*-value of 0.002) (Fig. 1).

### Course evaluation

A total of 117 participants (80%) completed the course evaluation. Participants thought the course was easy to navigate (mean 4.6 out of 5, SD 0.6), and effective (mean 4.6 out of 5, SD 0.6). Participants also reported that they would apply the information and incorporate strategies they learned to their work (mean 4.5 out of 5, SD 0.7), and were more likely to recommend HPV vaccination for eligible patients as a result of this course (mean 4.4 out of 5, SD 0.8). Finally, participants recommended this course (mean 4.5 out of 5, SD 0.7).

## Discussion

To our knowledge, our study is one of very few that compared HPV vaccination knowledge, attitudes, and self-reported behaviors by provider specialty. Given the low rates of HPV vaccination in the 18–26 and 27–45 age ranges, we specifically targeted providers at our institution who provided primary care for adult patients over 18 years of age, which included IM, FM, Med/Peds, and OB/GYN providers. It is significant that in our study, IM providers had lower baseline knowledge and lower confidence with regards to HPV vaccination compared to FM, Med/Peds, and OB/GYN providers. IM providers also self-reported that they spent less time counseling adult patients on HPV vaccination compared to FM, Med/Peds, and OB/GYN providers. Given the important role that IM providers play as primary care providers for the adult population, there is a strong educational opportunity to educate internists on the importance of HPV vaccination for adults. Studies show that a recommendation from a healthcare provider is the single best predictor of vaccination for any vaccine, including the HPV vaccine [16]. This underscores the need for internists and other providers delivering adult primary care to have comprehensive and actionable training to acquire the necessary knowledge to provide quality counseling to patients for HPV vaccination.

Though HPV vaccination was expanded for patients aged 27–45 years of age, rates of HPV vaccination in this age range remains low [8]. Our study further showed that across all specialties, providers were the least confident in their ability to identify patients aged 27–45 who needed HPV vaccination, and also discussed HPV vaccination less frequently in this age group when compared to patients aged 19–26. This further supports the importance to specifically target educational efforts for providers who provide care for adult patients on how to identify patients aged 27–45 who may benefit from shared clinical decision-making with regards to the HPV vaccination.

Currently, educational materials and resources on HPV vaccination are targeted more towards patients than healthcare providers. The quality, efficacy, and utilization of educational resources available for providers on HPV vaccination remains generally unknown [12]. Our study showed that an asynchronous online module was well received by busy providers and effective at improving HPV vaccination knowledge and confidence in identify patients who qualified for the HPV vaccination, and ability to counsel patients on HPV vaccination. Importantly, the improvements in knowledge and confidence were retained over time. Furthermore, our study also demonstrated that an online e-learning module was effective at changing provider behavior with regards to HPV vaccination counseling. More providers reported discussing HPV vaccination with patients and counseling patients on the risks of HPV infection.

We are aware that technology-based education can offer distinct advantages and challenges. While the online format of this CME dovetailed with the growing technical skillset of clinicians and the need to be physically remote during the COVID-19 pandemic, there may have also been a saturation point for participants preferring to limit screen time in the setting of virtualized medicine, opting out of more virtual initiatives. Our module was therefore designed by applying the Six-Step Approach for curriculum development with an eye towards conciseness, usability and interactivity as strategies to encourage online user engagement. Research has shown that using this systematic framework while considering factors specific for online curriculum development can lead to more effective educational program development [17].

Important limitations must be considered when reviewing our work. First, administrative factors presented notable limitations to our study. An administrative error during the initial deployment of the module led to a gap in demographic and behavior data for 31 participants before the error was identified and corrected. Secondly, while the delayed post-test demonstrated significant knowledge retention over an eight-month period, interpretation is limited by a relatively small cohort of 46 respondents, or 32% of the initial participants. Clinician turnover and general clinician bandwidth and availability during the COVID-19 pandemic were likely factors in this lower response rate. We also did not assess HPV counseling skills for this asynchronous module, but hope to assess this in the future.

Finally, our education module was developed, deployed, and analyzed during the height of the COVID-19 pandemic, during which there was a significant reduction in in-office care and consequently, a reduction in routine vaccinations including HPV vaccination. An initial study by Daniels et al. showed that HPV vaccination rates among individuals aged 9–26 in March and April of 2020 were 23% of the previous year’s rate, and only reached 48% of the previous year’s rate by August 2020 [18]. Moreover, as the COVID-19 vaccine became available, initial guidance from the CDC actively prioritized patient counseling for the COVID-19 vaccine. We hypothesize that providers likely did not have the bandwidth to engage in HPV vaccine counseling after concluding a COVID-19 vaccine counseling session, and thus, HPV vaccine counseling likely naturally reduced during this time period. Unfortunately, these unique environmental factors thus impeded our ability to compare patient vaccination rates without considering a bevy of confounding variables.

Future directions for our study include analyzing patient level data to determine whether increased confidence and knowledge of providers translated into higher rates of HPV vaccination for patients.

## Conclusion

The goal of this educational module was to improve confidence and knowledge of HPV vaccination among adult primary care providers and to encourage providers to counsel eligible patients on HPV vaccination. We chose to create a short, asynchronous, e-learning module that was easily accessible and well-received by busy providers. This study highlighted that an online educational module was effective at improving HPV vaccination confidence, knowledge, and self-reported behavior, and addressed a need for HPV education for providers. Given the important role that healthcare providers play in vaccine uptake, we believe that a provider-specific online educational intervention can be a powerful tool to encourage increased utilization and delivery of the HPV vaccine. Furthermore, with the lower rates of HPV vaccination in adults over 18, we believe further efforts are needed to educate internists and other providers who take care of the adult population on HPV vaccination. Currently, our module is not available outside of our institution. In the future, we hope to evaluate the effect of this educational module on HPV vaccination rates for our patients and disseminate our educational module to a broader audience.

## Data Availability

The datasets used and/or analyzed during the current study are available from the corresponding author on reasonable request.

## Abbreviations

HPV: Human papillomavirus
FDA: Food and Drug Administration
PCPs: Primary care providers
IM: Internal medicine
Med/Peds: Medicine/pediatrics
OB/GYN: Obstetrics/gynecology
FM: Family medicine
MDs: Medical doctors
Dos: Doctors of osteopathic medicine
NPs: Nurse practitioners
CNMs: Certified nurse midwives
PAs: Physician assistants
MOC: Maintenance of Certification
CME: Continuing Medical Education
SD: Standard Deviation
